# Regulatory Science: exploring an emerging scientific discipline in health care

**DOI:** 10.3389/fmed.2026.1837248

**Published:** 2026-06-15

**Authors:** Laura Volpi, Michael D’Agosto, Tom Melvin, Barbara Sickmüller, Julian S. Thimm

**Affiliations:** 1 Heidelberg University Hospital, Hospital Pharmacy, Heidelberg, Germany; 2 Faculty of Engineering and Technology, Furtwangen University, Tuttlingen, Germany; 3 Institute for Clinical Trials, College of Medicine, Nursing and Health Sciences, University of Galway, Galway, Ireland; 4 Deutsche Gesellschaft für Regulatorische Angelegenheiten e.V. (DGRA) - German Society of Regulatory Affairs, Bonn, Germany; 5 GMP and T Cell Therapy Group, German Cancer Research Center (DKFZ), Heidelberg, Germany

**Keywords:** decision making, medical device legislation, pharmaceutical legislation, public health, regulation, science

## Abstract

Regulatory Science has gained increasing prominence in the regulatory community, yet its character as a scientific discipline remains insufficiently clarified. While the term is widely used to describe activities that support regulatory decision-making, a unified normative anchoring is lacking. Existing definitions vary considerably in scope, often reflecting specific institutional contexts rather than a coherent scientific framework. This perspective examines Regulatory Science by reflecting on its scientific characteristics, intention, and potential contribution to effective regulation in the European health system. The article explores Regulatory Science as an emerging discipline by applying a humanity-based approach of conceptually scrutinizing its scientific core. By integrating insights from existing, non-congruent definitions, current activities, and regulatory practice, this perspective argues that Regulatory Science extends beyond compliance-oriented regulatory activities and product-specific assessment. Instead, it is positioned as a scientific approach to systematic, evidence-based reflection on regulatory systems, their functioning, and their further development in response to real market needs. Regulatory Science is proposed to provide the methodological foundation for meaningful regulation, consistent with its recitals that provide the objective for regulatory requirements. Based on this reflection, the article postulates conditions under which Regulatory Science can be justified as a distinct scientific discipline. It contributes to its further constitution in the scientific field through highlighting a required normative structure. This results in the proposal of a new, refined definition emphasizing regulation itself as the research objective, the need for scientific methodology, and its overall intention.

## 1 Introduction

The regulatory framework governing health care products has become increasingly complex, given their impact on public health in terms of both benefits and risks. In response to this complexity, the term Regulatory Science has gained prominence in policy-oriented discourse, e.g., resulting in the launch of the European Platform for Regulatory Science Research (1). It is described as activities intended to support regulatory decision-making and the evaluation of health products, aiming to overcome gaps between regulation and scientific progress (2). Despite its widespread adoption, Regulatory Science lacks a consistently applied and scientifically grounded definition (3). Consequently, the term Regulatory Science is frequently used to denote a collection of practices or objectives, rather than to describe a coherent scientific discipline with a clearly articulated epistemic basis (4–7).

The broad and heterogeneous use of the term further contributes to conceptual ambiguity, reinforcing the need for critical reflection on whether Regulatory Science can be meaningfully characterized as a scientific discipline on its own. An unspecific, general understanding of Regulatory Science hinders the establishment of this scientific discipline as a valuable contribution to science. Without a precise scope that reflects actual needs, there is no foundation for developing scientific conventions or value to the regulatory field.

This study addresses the discipline’s current state of development by conceptually examining the normative structure of Regulatory Science. The overall aim is to provide the regulatory scientific community with a viewpoint on relevant regulatory objectives to work in accordance with scientific standards that provide sufficient scientific rigor.

### 1.1 Preconception of science and regulation

The connection between science and regulation is not an entirely new concept: Regulatory frameworks have long acknowledged the role of scientific evidence in informing regulatory decision-making, as exemplified by Recital No. 85 of Regulation (EU) 2017/745 on medical devices (MDR), which states that the European Commission should ensure the effective and uniform implementation of regulation based on sound scientific evidence (8). For centrally authorized medicinal products Art. 5 and 6 of Regulation (EC) No 726/2004 constitute the Committee for Medicinal Products for Human Use (CHMP) as a scientific assessment body for regulatory evaluation (9). In parallel, according to Art. 1 of their rules of procedure, the Coordination group for Mutual Recognition and Decentralized Procedures – human (CMDh) is required to maintain scientific expertise in its regulatory activities, coordinating decentralized marketing authorization procedures (10). Such references underscore a learning from historic regulatory decision-making that lacks scientific and clinical reasoning (11).

The increased complexity of regulation becomes particularly apparent in early development and translational phases, where regulatory considerations increasingly shape scientific and development strategies, determining the feasibility of novel therapies (12). In the European Union, this is currently also reflected in several revisions and amendments, such as for the EU pharmaceutical legislation, the European Biotech Act, the Medical Device Regulation, the *In Vitro* Diagnostic Medical Device Regulation, and the EU AI Act (13–16).

At the same time, the translation of (new) medical interventions into the market is increasingly important as the medical sector faces severe challenges regarding the availability and economic sustainability of therapy options in medical care (17). Therefore, the responsibility of the regulatory framework in health care is not only to provide feasible market access for health products, but also to ensure sufficient access to treatment for patients.

However, implementing regulatory expertise into these development stages remains a challenge. For that reason, EMA has formed the innovation task force (ITF) for targeted early engagement dialog available for all types of developers, from academic to large companies (18). Initiatives such as the Innovative Health Initiative (IHI) by the European Union in partnership with the health industry aim to translate scientific research into compliance-relevant evidence and collaborative frameworks (19).

Measures such as these are particularly important for new medical concepts such as advanced therapy medicinal products (ATMPs), personalized therapies, or breakthrough devices as they face severe regulatory challenges with regard to the evaluation of market readiness (20, 21). Additionally, AI-enabled medical devices face overlapping requirements and limited guidance (22).

Against this background, Regulatory Science is initially approached in this perspective as aiming to close the gap between existing regulation and the rapidly evolving outcomes of medical research. According to this preconception, fostering regulatory exchange among stakeholders is supposed to promote the translation of advanced medical solutions into marketed products.

## 2 Analytical approach and subjects covered

To reflect the normative structure underlying Regulatory Science, an epistemological approach of hermeneutics is applied. Within humanities, hermeneutics is commonly applied to understand meaning and intention – focusing on context rather than empirical quantification (23). It allows different conceptual starting points and views on Regulatory Science to be critically examined, rather than applying a restrictive systematic review logic. For that, the hermeneutic cycle provides an analytical framework to iteratively refine understanding by synthesizing thematic conclusions in cycles, which are subsequently critically reflected and integrated into the overall argument.

**Figure 1:**
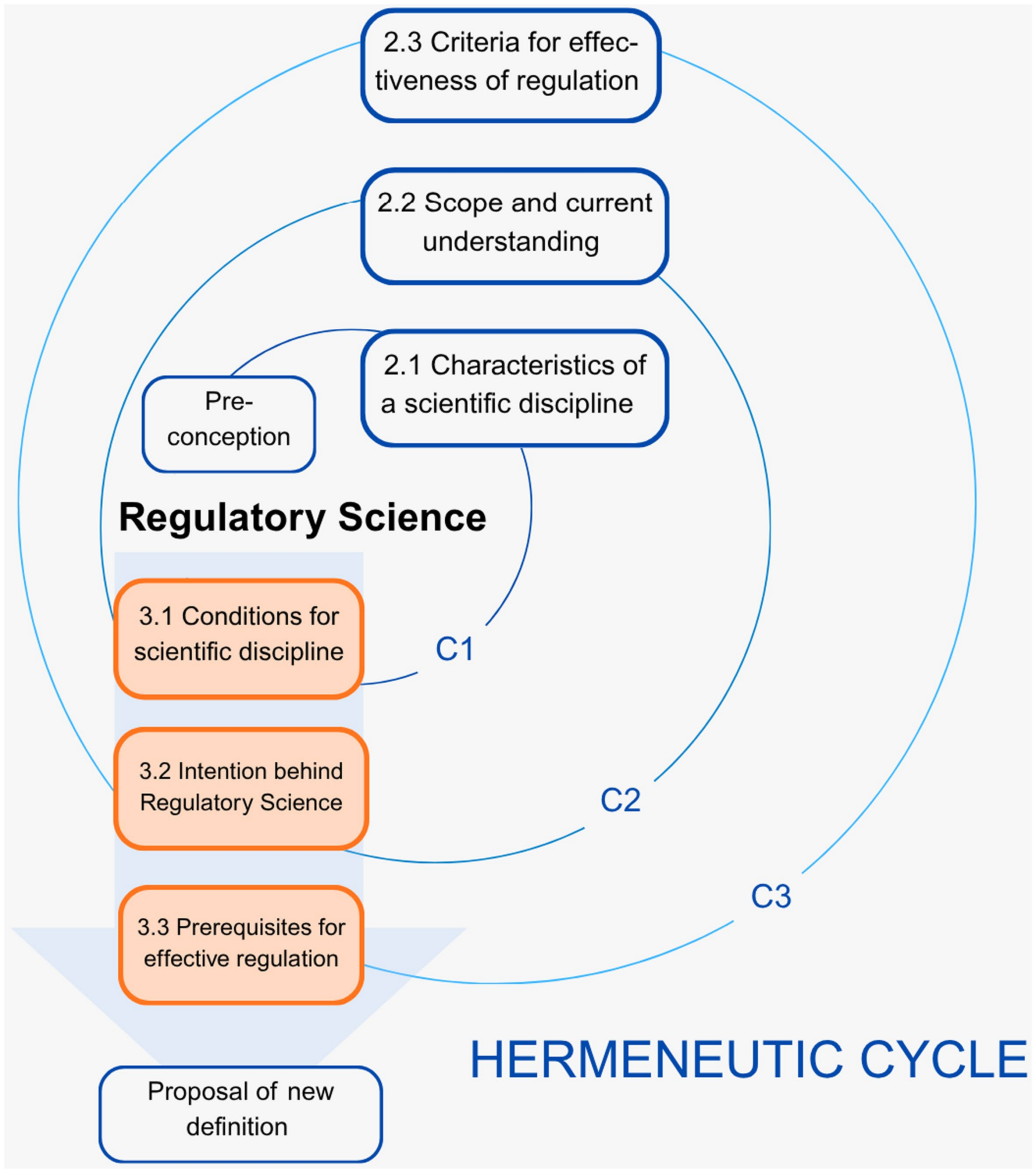
Graphical display of the hermeneutic approach as layered circles around the central topic of Regulatory Science. This figure was created using Canva.

Accordingly, this perspective adopts a hermeneutic approach, entering the cycle based on a preconception outlined in the introduction. Because of the question of the scientific value of Regulatory Science, the reflection was first guided by the overarching characteristics of a scientific discipline (C1). Further, to assess the intention behind Regulatory Science, the view was directed at the current understanding represented in collected term descriptions, as well as work already conducted (C2). Finally, to reveal the true need for Regulatory Science, the question of prerequisites for effective regulation was addressed (C3). Figure 1 illustrates the iterative analytical process of a hermeneutic cycle.

The identified sources for interpretation are each outlined in the following sections. For each topic, sources were selected through a conceptually guided, relevance-based literature search, meaning that their inclusion was determined by their contribution to the respective analytical question.

### 2.1 Characteristics of a scientific discipline

Whether Regulatory Science can be regarded as a scientific discipline depends on how core characteristics of scientific work are applied in this context. From this viewpoint, Münster’s definition provides a common perspective on “science” within epistemology (24). It emphasizes the generation of knowledge beyond individual cases and the identification of generalizability as key identifiers.

Furthermore, scientific character is anchored in systematic methodology that is reflected in general principles of Good Scientific Practice (GSP), which define foundational standards for the generation, documentation, and communication of scientific knowledge (25). For the regulatory domain the World Health Organization (WHO) published the Good Regulatory Practice (GRP) principles that set high-level ground rules for their member states to support a well-performing regulatory framework for medical products (26). These principles aim to support sound and effective regulation with a focus on public health, patient protection, and evidence-based decision-making.

### 2.2 Scope and current understanding of Regulatory Science

Various institutions globally published definitions or characterizations of the term “Regulatory Science.” An overview of all retrieved descriptions is provided in Supplementary Table S1. Existing definitions/ descriptions were categorized by stakeholder groups to further address the heterogeneity of angles and potential institutional intentions for the need of a scientific discipline.

Furthermore, the study investigates how Regulatory Science is currently applied in academic work. For that, the specific activities of the institutions that positioned themselves by providing a definition or description were examined; in addition, the deliberate academic activities by the German Society of Regulatory Affairs (DGRA) within the master’s study program and the work by the Berlin Center for Gene and Cell Therapy were examined (27, 28). This examination focused on underlying trends to identify actual needs in this domain and, based on that, to extract the scope of a scientific discipline.

### 2.3 Criteria for effectiveness of regulation

Based on the insights into the current normative situation of Regulatory Science gained through C1 and C2 of the hermeneutic cycle, the topic was expanded in the final step to include the issue of effective regulation. For that, the regulatory theory by the German constitutional lawyer Georg Jellinek “Theory of the State,” was examined: According to this theory, there is a correlation between regulatory intent and practical application: Regulation is not only developed through deliberate placement but also through recognition of the practical reality (29). The better the fit between the two, the more likely the regulation is to be accepted and therefore functional. Acceptance and fit with real-world practice are key drivers of the function of regulation, as intended in the specific recitals.

This theory on fundamental criteria for effective regulation was used to form a viewpoint on the overall aim of Regulatory Science at the epistemic basis.

## 3 Discussion

The results of the hermeneutic reflection on Regulatory Science have provided extensive insight into the epistemological reasoning as a distinct scientific discipline:

Provided that regulation is intended to fit normative standards appropriatly, Regulatory Science may contribute to a well-functioning, feasible regulatory system. Examining discrepancies between regulatory intent and factual implementation—both under- and over-regulation—can provide insights into the effectiveness and sustainability of regulatory frameworks. Accordingly, its contribution lies in supporting evidence-based regulatory decision-making and policy development. In this sense, Regulatory Science provides a means to assess whether regulatory rigor translates into proportional patient benefit and societal value.

The following sections provide a more detailed breakdown of how this final conception was developed.

### 3.1 Conditions for scientific discipline

**Figure 2:**
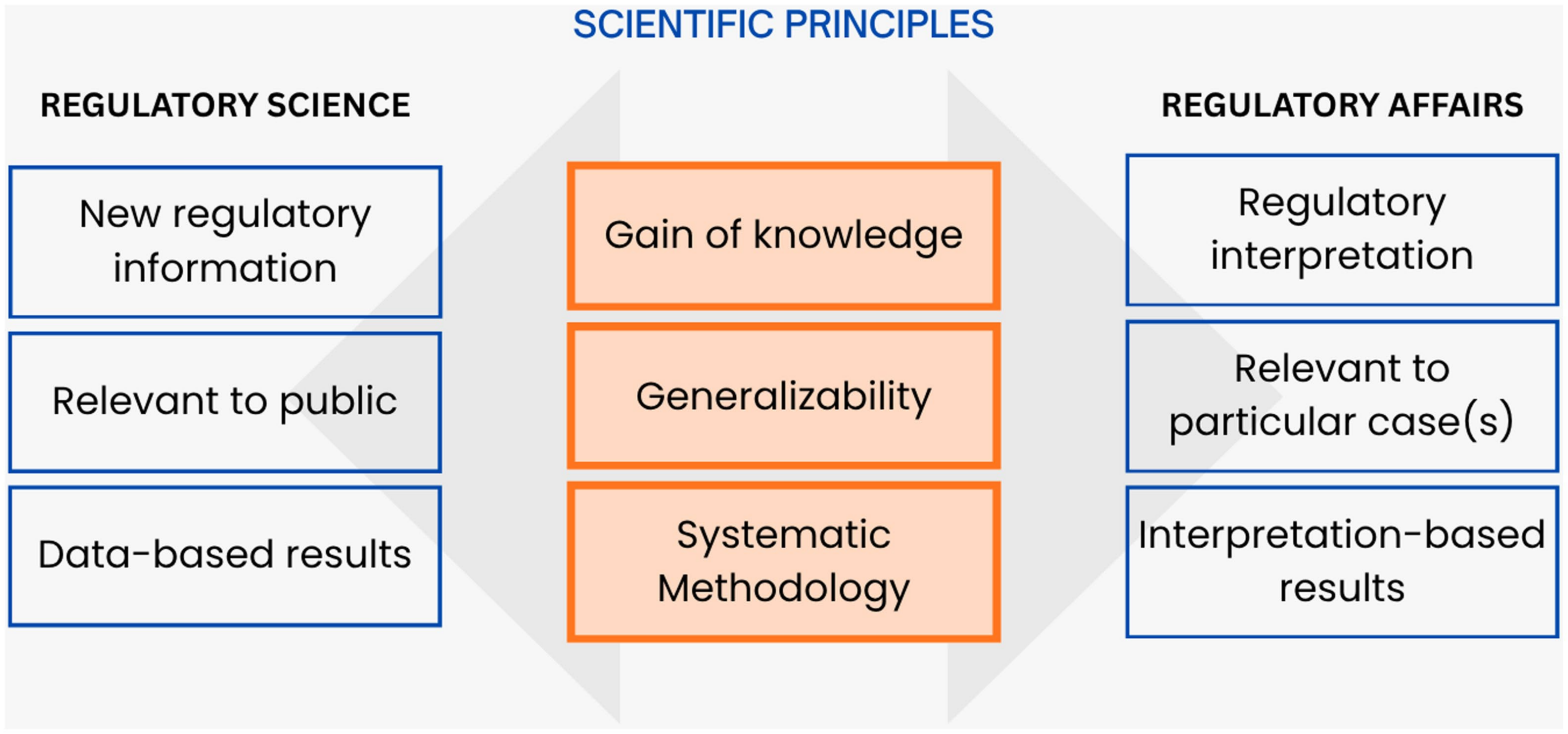
Overview of scientific principles used to differentiate between Regulatory Science and Regulatory Affairs. This figure was created using Canva.

First, to establish Regulatory Science as an independent scientific discipline, it must be linked to fundamental scientific characteristics as set out by Münster. A distinction from regulatory affairs management is required, which primarily focuses on product-specific implementation of regulatory requirements to ensure compliance (see Figure 2).

If Regulatory Science is understood as a scientific aspiration, the results should be obtained using systematic, reliable methods, as set out by the German Research Foundation, for example. According to that, in the responsibility of both organizations and researchers themselves, the commitment to these general principles is supposed to ensure scientific integrity for trustworthy research (25).

In parallel, Good Regulatory Practice (GRP) by WHO describes a science and data-driven regulatory decision-making process as a key GRP enabler (26). This enabler is rooted in GRP principles such as transparency or proportionality. To provide evidence that these principles are being well executed during policy making, the collection of data in a scientifically sound, objective manner can be an asset. For that, Regulatory Science can fulfill the purpose of supporting regulatory decision-making—provided appropriate scientific conventions are established with the increasing recognition as a scientific discipline.

### 3.2 Intention behind Regulatory Science

Overall, it is noteworthy that a variety of stakeholders – including authorities – support the concept of Regulatory Science, highlighting the scientific need despite the absence of a universal scope. According to EMA’s updated description of Regulatory Science in the concept paper on the medicines landscape, there is an aspiration to connect academic medical research to regulatory support (30). Similarly, the definition by ABPI as an industry association highlights the translation of new technologies into available treatment options. The categorization of stakeholders reveals a strong focus by authorities and industry representatives on Regulatory Science as a discipline to promote the availability of novel therapy options, mainly through the connection of research / academia to regulators.

While stakeholder interaction in light of this perspective functions as an enabling element, according to general scientific principles, it can not constitute the only scientific core of the discipline.

As research institutes such as the Danish CORS focus more on further development of regulatory requirements, the intention behind Regulatory Science as a contribution to regulatory systems is concluded. For that, a potential role not only in the evaluation of downstream requirements, but as an integral component in the regulatory framework shaping the translation of new medical interventions into regulation is seen.

In current scientific work in the regulatory domain, multiple levels of scientific inquiry were observed. From retrospective analysis of regulatory processes at a metalevel to new evaluation methods for assessing market product readiness, a variety of objectives are being pursued. EMA also deliberately published Regulatory Science Research Needs within seven research domains, which reflect a broad scope beyond natural sciences (31). It is noted that without a commonly accepted understanding of Regulatory Sciences vision, the classification of relevant research to the field is difficult.

Keeping the scientific core principles in mind, relevant sources for regulatory research were considered. Although health technology assessments are based on scientific principles, these efforts are not directly considered Regulatory Science, as the research subject is a certain medical intervention. Similarly, advice scenarios or structured dialogs acc. to MDCG 2019–6 Rev. 5 between manufacturers and authorities/notified bodies: These are targeted consultation settings for a specific product approval route or conformity assessment (within that often-concerning clinical strategies) and therefore for intellectual property reasons not immediately available to the public, if at all. Especially, the medical device sector lacks transparency regarding conformity assessments. However, results from procedures to obtain market readiness, such as scientific advice, European Public Assessment Reports, or Clinical/Performance Evaluation Consultation Procedure, could be a reliable source of information for the specific assessment of regulation.

The overarching objective behind these efforts was seen as the need for a highly functional regulatory system that is further developed based on approaches supported by scientific evidence. At the same time, the availability of medically necessary products in the market should be ensured by the regulatory system, promoting the smooth functioning of the internal market, as generally set out in Article 26 of the Treaty on the Functioning of the European Union. This is reflected in several regulations on medical products, such as No. 2 of Regulation (EU) 2017/745 or No. 2 of Directive 2001/83/EC. Ultimately, it seems that at the core of Regulatory Science stands the need for sensible, effective regulation to ensure safe healthcare products and to contribute to adequate, functional medical care.

### 3.3 Prerequisites for effective regulation

According to Jelinek’s theory of state, the effectiveness of regulation is closely linked to its acceptance and practical feasibility within the healthcare system. This is—through regulatory recitals—centered on goals of protection for public health, patient safety, and the availability of medical interventions in the market. In light of high financial stakes to bring products to market, and at the same time, uncertainty of return on investment (32, 33), it seems logical that the benefits of strict regulatory requirements should be questioned objectively. It is reflected that high product requirements can result in caution by manufacturers to bring necessary products into the market, especially when high stakes do not align with the regulations’ intention for true patient protection. This becomes apparent in increased shortage situations in the pharmaceutical market as well as withdrawal of device manufacturers from the market due to potentially disproportionate MDR requirements (34, 35). As a result, there is a risk of contradicting the recitals regarding unhindered industry development and trade stated in recital No. 3 of the Community Code on Medicinal Products or recital No. 2 of the Medical Device Regulation (8, 36).

Taken together, the preceding discussion illustrates that Regulatory Science cannot be reduced to a collection of regulatory tools or supportive activities; rather, it represents an emerging scientific approach that systematically examines regulation itself as an object of inquiry. There is the opportunity to position Regulation Science as a neutral, evidence-based contributor to regulatory development, complementing the roles of legislators and regulatory authorities—particularly in the context of rapidly evolving healthcare technologies. This perspective on Regulatory Science is also reflected in the right to good administration anchored in Art. 41 of the European Charter of Fundamental Rights and the European Commission’s initiative of “Better Regulation Guidelines,” which both shall contribute to highly functional regulation to serve the public (37–39).

At the same time, the absence of a consolidated understanding of its scope, methodological foundations, and disciplinary boundaries highlights that Regulatory Science remains in a formative stage. This underscores the need for continued conceptual clarification and provides the basis for further reflection on how Regulatory Science may be defined and positioned as a scientific discipline. A neutral stakeholder between regulatory considerations and scientific evidence can be beneficial to reveal true clinical and market needs for regulation, not just industry needs.

## 4 Proposal for a new definition

Considering the issues discussed above, this perspective article illustrates that the normative background of Regulatory Science remains unclear. The current use of the term obscures the absence of a commonly accepted normative foundation. Through this study, a broader scientific potential is indicated, extending beyond product- and compliance-centered regulatory activities. This gained understanding contributes to the development of a normative structure for Regulatory Science by proposing a new definition:

“Regulatory Science is an interdisciplinary science, that focusses on regulation itself as its research objective by contributing to analysis, evaluation and (further) development of regulatory requirements and methods of compliance based on scientific methodologies assuring scientific evidence for objective evaluation.

It is not limited to research on regulation for novel therapy approaches but questions the overall suitability of health care regulation according to its intentions.

Regulatory Science uses reliable sources of data that allow insight into the true function of the regulatory system and contributes to scientific discourse.”

## Data Availability

The original contributions presented in the study are included in the article/Supplementary material, further inquiries can be directed to the corresponding author.
