# Supplementary material for: Regulatory Science: exploring an emerging scientific discipline in health care
Source: Front Med (Lausanne). 2026 Jun 15;13:1837248. doi: 10.3389/fmed.2026.1837248 (PMC13310879; doi:10.3389/fmed.2026.1837248)
Supplement: Supplementary file 1 [file Data_Sheet_1.docx]

Supplementary Material

# Supplementary Figures and Tables

Table 1 Overview of gathered definitions of the term “Regulatory Science” with assignment of the respective institution to a stakeholder category. The stakeholders A, B, D, G and H provided different definitions over the preparation period – the respective second definition is the current one.

| # | Institution | Stakeholder | Definitions and Visions of Regulatory Science |
| --- | --- | --- | --- |
| A.1 | European Medicines Agency (EMA) | Regulatory Agency | „‘Regulatory science’ refers to the range of scientific disciplines that are applied to the quality, safety and efficacy assessment of medicinal products and that inform regulatory decision-making throughout the lifecycle of a medicine. It encompasses basic and applied biomedical and social sciences and contributes to the development of regulatory standards and tools.”(1) |
| A.2 | European Medicines Agency (EMA) / Heads of Medicines Agencies (HMA) | Regulatory Agency | “Regulatory science plays a critical role in public health by bridging the gap between scientific research and the practical application of that research in regulatory decision making.” (2) |
| B.1 | Food and Drug Administration (FDA) | Regulatory Authority | „Regulatory Science is the science of developing new tools, standards, and approaches to assess the safety, efficacy, quality, and performance of all FDA-regulated products.”(3) |
| B.2 | Food and Drug Administration (FDA) | Regulatory Authority | „Regulatory Science is a field of scientific research focused on developing the tools, standards, and approaches needed to generate the data and information on which FDA assesses the safety, efficacy, quality, and performance of FDA-regulated products. Advances in regulatory science are critical to support regulatory decision-making and to help enable the translation of discoveries in science and technology into safe and effective medical products.“ (4) |
| C | Center of Drug Evaluation and Research (CDER) at FDA | Regulatory Authority | „Regulatory science at FDA is the science of developing new tools, standards, and approaches to assess the safety and performance of the products it regulates. CDER's regulatory science activities are aimed at speeding the development of new drugs while ensuring that they are safe and effective.“(5) |
| D | Association of the British Pharmaceutical Industry (ABPI) | Industry Association | Regulatory science helps bridge the gap between research and policy, ensuring that the latest technologies can be translated into safe and effective treatments for people and patients.(6) |
| E | Copenhagen Center of Regulatory Science (CORS) –  University of Copenhagen | Research Institute | We define Regulatory Science as the science that informs, facilitates and/or evaluates regulatory decision making.  Drug Regulatory Science is applied to medicincal products and concerns:   - Evaluating the performance of drug regulations and regulatory instruments; - Developing tools and methods to support regulatory decision-making; and - Producing evidence informing regulatory decision-making   Mission  Through research and education in regulatory science, CORS will improve the drug regulatory system and thereby contribute to an improvement of the health of the society and sustainable drug innovation.    With ambitions in the following subdomains:   - Methodological research: Strengthening Regulatory Science as an established scientific discipline - Applied regulatory science: Stimulating a sustainable regulatory system, by systematically studying its structure and behavior, as well as designing new tools to facilitate regulatory decision-making. - Valorisation: Increasing societal relevance of research by utilizing the inter-disciplinary and cross-sectional setup of CORS. - Education: Cultivating the regulatory environment for pharmaceutical innovations and emerging technologies.(7) |
| F | UCSF-Stanford Center of Excellence in Regulatory Science and Innovation (CERSI) | Network Organisation | The UCSF-Stanford Center of Excellence in Regulatory Science and Innovation (UCSF-Stanford CERSI) is a joint undertaking among:   - University of California, San Francisco (UCSF), School of Pharmacy and School of Medicine - Stanford University, School of Engineering and School of Medicine - U.S. Food and Drug Administration (FDA)   These three partners work collaboratively on projects that promote the emerging field of regulatory science—including innovative research, education, outreach, and scientific exchange—together with foundations and commercial entities interested in the development of FDA-approved medical products. Collaborations between the center and the pharmaceutical, biotechnology, and high-tech industries of the San Francisco Bay Area and the West Coast are especially important to the center’s aims.  The center works closely with FDA members and provides modern technology tools that will help evaluate medical products for safety, efficacy, quality, and performance. (8) |
| G.1 | Center for Innovation in Regulatory Science (CIRS) | Network Organization | CIRS’ mission:  „To maintain a leadership role in identifying and applying scientific principles for the purpose of advancing regulatory and health technology assessment (HTA) policies and processes in developing and facilitating access to pharmaceutical products. (9) |
| G.2 | Center for Innovation in Regulatory Science (CIRS) | Network Organization | CIRS’ mission:  „Identify and apply scientific principles for the purpose of advancing regulatory and health technology assessment (HTA) policies and processes in developing and facilitating access to pharmaceutical products.“(10) |
| H.1 | Else Kröner Fresenius Center for Digital Health, Dresden University of Technology | Research Institute | “My research goal is to advance regulatory requirements, especially for software as a medical device and artificial intelligence in medical devices. Innovative approaches to healthcare of the future must be accompanied by innovative approaches to regulation“ – Quote by Prof. Dr. Stephen Gilbert (11) |
| H.2 | Else Kröner Fresenius Center for Digital Health, Dresden University of Technology | Research Institute | “Our aim is to rethink the regulation of medical devices. Tomorrow’s innovative healthcare solutions require innovative regulatory approaches.“ (12) |
| I | Fraunhofer IMTE – Fraunhofer Research Institution for Individualized and Cell-Based Medical Engineering | Research Institute | „The innovative power of medical engineering also requires a high degree of reactivity with regard to regulatory aspects. Accordingly, existing regulatory requirements are critically scrutinized at Fraunhofer IMTE and questioned for their applicability to innovative product areas. The scientific conception and further development of corresponding strategies represents a focal point in order to always be able to guarantee the safety, performance and effectiveness of medical products.“ (13) |
| J | Johner Institut GmbH | Regulatory Consultant | Translated: „Regulatory Science” is a science. It develops the (technical) foundations, processes, methods and tools,   - To formulate regulatory requirements that ensure the safety, performance and efficacy of medical devices, and - To understand and anticipate the economic and other impacts of these regulatory requirements on healthcare systems and the economy.“ (14) |
| K | Faculty of Engineering & Technology, Furtwangen University | Research Institute | Translated: “[Regulatory] Science has a scientific approach to it – in other words, it is also about gaining knowledge. How we can understand how such new regulatory systems function and work. And, of course how we can then design them as the next step. For example, how we can build new regulatory systems, how we can optimize them and how we can develop new approaches.”  Quote by Prof. Dr. Martin Haimerl (15) |

**References**

1. European Medicines Agency. EMA Regulatory Science to 2025 - Strategic reflection [Internet]. Amsterdam; 2020 [cited 2025 May 18]. Report No.: EMA/110706/2020. Available from: https://www.ema.europa.eu/en/documents/regulatory-procedural-guideline/ema-regulatory-science-2025-strategic-reflection_en.pdf

2. European Medicines Agency. Seizing opportunities in a changing medicines landscape - The European medicines agencies network strategy 2028 [Internet]. Luxembourg: Publications Office of the European Union; 2025. Report No.: TC-01-25-020-EN. Available from: https://www.ema.europa.eu/en/documents/other/seizing-opportunities-changing-medicines-landscape-european-medicines-agencies-network-strategy-2028-final_en.pdf doi:doi:10.2809/8994389

3. Food and Drug Administration. Advancing Regulatory Science [Internet]. [cited 2024 Jul 17]. Available from: https://web.archive.org/web/20240717043632/https://www.fda.gov/science-research/science-and-research-special-topics/advancing-regulatory-science

4. Food and Drug Administration. Advancing Regulatory Science - Regulatory Science Programs [Internet]. FDA; 2024 [cited 2025 Jun 16]. Available from: https://www.fda.gov/science-research/science-and-research-special-topics/advancing-regulatory-science

5. Center for Drug Evaluation and Research. Regulatory Science at CDER [Internet]. 2024 [cited 2025 Jun 16]. Available from: https://www.fda.gov/drugs/science-and-research-drugs/regulatory-science-cder

6. Association of the British Pharmaceutical Industry. Regulation and Regulatory Science [Internet]. [date unknown] [cited 2025 Jun 16]. Available from: https://www.abpi.org.uk/r-d-manufacturing/regulation-and-regulatory-science/

7. Copenhagen Centre for Regulatory Science. About the Copenhagen Centre for Regulatory Science [Internet]. University of Copenhagen; 2018 [cited 2025 Jun 16]. Available from: https://cors.ku.dk/about

8. University of California San Francisco. UCSF-Stanford Center of Excellence in Regulatory Science and Innovation (CERSI) [Internet]. [date unknown] [cited 2025 Jun 16]. Available from: https://pharm.ucsf.edu/cersi

9. Centre for Innovation in Regulatory Science. About us - Centre for Innovation in Regulatory Science: CIRS [Internet]. [cited 2024 Jul 21]. Available from: https://web.archive.org/web/20240721141522/https://www.cirsci.org/about-us/

10. Centre for Innovation in Regulatory Science. About us - Centre for Innovation in Regulatory Science: CIRS [Internet]. 2020 Sep 7 [cited 2025 Jun 16]. Available from: https://www.cirsci.org/about-us/

11. Else Kröner Fresenius Center for Digital Health. Medical Device Regulatory Science [Internet]. [cited 2025 Jun 16]. Available from: https://web.archive.org/web/20240703061719/https://digitalhealth.tu-dresden.de/people/regulatory-science/

12. Else Kröner Fresenius Center for Digital Health. Medical Device Regulatory Science [Internet]. [cited 2025 Jun 16]. Available from: https://digitalhealth.tu-dresden.de/people/regulatory-science/

13. Fraunhofer Research Institution for Individualized and Cell-Based Medical Engineering. Regulatory Science [Internet]. [cited 2025 Jun 16]. Available from: https://www.imte.fraunhofer.de/en/researchfields/regulatory-science.html

14. Johner C. Regulatory Science: Europa im Blindflug [Internet]. 2021 Jun 28 [cited 2025 Jun 16]. Available from: https://www.johner-institut.de/blog/regulatory-affairs/regulatory-science/

15. Haimerl M. Regulatory Science - Why we need this science. Medical Device Insights [Internet]. 2021. Available from: https://johner-institut.podigee.io/21-regulatory-science
